# Synthesis of three-dimensionally interconnected sulfur-rich polymers for cathode materials of high-rate lithium–sulfur batteries

**DOI:** 10.1038/ncomms8278

**Published:** 2015-06-12

**Authors:** Hoon Kim, Joungphil Lee, Hyungmin Ahn, Onnuri Kim, Moon Jeong Park

**Affiliations:** 1 Department of Chemistry, Pohang University of Science and Technology (POSTECH), Pohang 790-784, Korea; 2 Division of Advanced Materials Science, Pohang University of Science and Technology (POSTECH), Pohang 790-784, Korea

## Abstract

Elemental sulfur is one of the most attractive cathode active materials in lithium batteries because of its high theoretical specific capacity. Despite the positive aspect, lithium–sulfur batteries have suffered from severe capacity fading and limited rate capability. Here we report facile large-scale synthesis of a class of organosulfur compounds that could open a new chapter in designing cathode materials to advance lithium–sulfur battery technologies. Porous trithiocyanuric acid crystals are synthesized for use as a soft template, where the ring-opening polymerization of elemental sulfur takes place along the thiol surfaces to create three-dimensionally interconnected sulfur-rich phases. Our lithium–sulfur cells display discharge capacity of 945 mAh g^−1^ after 100 cycles at 0.2 C with high-capacity retention of 92%, as well as lifetimes of 450 cycles. Particularly, the organized amine groups in the crystals increase Li^+^-ion transfer rate, affording a rate performance of 1210, mAh g^−1^ at 0.1 C and 730 mAh g^−1^ at 5 C.

## Abstract (web summary)

There is intensive research underway into the cathode development of lithium–sulphur batteries. Here, the authors present a battery with organosulfur-containing polymers as the cathode active materials which displays promising electrochemical performance.

## Graphical abstract

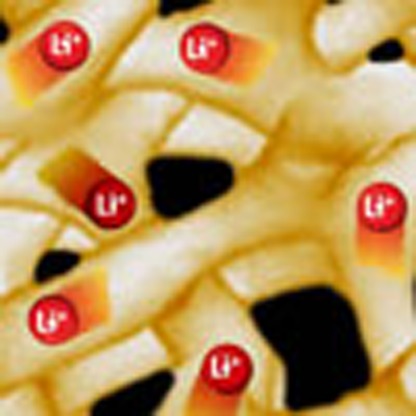

As lithium-ion batteries (LIBs) become more common in our daily lives, the demand for high-energy-density LIB for use in emerging large-scale energy storage systems and electric vehicles, as well as in small appliances, has increased. Therefore, the development of new electrode active materials beyond the graphite^1^,^2^,^3^ and lithium metal oxides^4^,^5^ used in currently established LIBs is inevitable. Considerable attention has thus been paid to lithium–sulfur (Li–S) batteries that use elemental sulfur as a cathode active material^6^,^7^,^8^ since the Li–S cells can deliver a fivefold higher energy density than conventional LIBs by taking advantage of the high specific capacity of sulfur (∼1,675 mAh g^−1^)^9^,^10^. The light weight, low cost, natural abundance and environmentally benign nature of sulfur are also desirable properties that make it suitable for application in future energy materials^11^,^12^.

Despite the above-mentioned benefits of sulfur, Li–S batteries have major drawbacks such as poor long-term performance^13^ and limited rate capability^14^. Significant past research has shown that the dissolution of lithium polysulfides and the inherently insulating nature of sulfur to both electrons and lithium ions^15^,^16^,^17^ are responsible for these obstacles. The large volume expansion of sulfur, by up to 80% on full lithiation, has also been identified as an important factor that should be considered in sulfur electrode design^18^.

Various methods to resolve these challenges have been reported over the past decade. Major improvements, inspired by the work of Nazar *et al*.^19^ have been made by exploiting structured carbon materials that can physically confine sulfur to mitigate the shuttling of polysulfides, accompanied with facilitated fast electron transfer^18^,^19^,^20^,^21^,^22^. Variation of the type of carbon, by use of porous carbon^23^,^24^,^25^, hollow carbon spheres^7^,^26^,^27^, carbon nanotubes/nanofibres^28^ and graphene/graphene oxide^29^,^30^,^31^, helps in achieving long cycle life (>300 cycles), improved capacity retention (∼70 %) and high Coulombic efficiency^6^,^7^,^32^. Nevertheless, a gradual decrease in capacity with prolonged battery cycling is still observed. This decrease in capacity is a consequence of the low binding energy between sulfur and carbon matrices, and has prompted recent efforts to intensify specific attractions between sulfur and carbon frameworks via surface modification of carbon^33^,^34^,^35^.

While the use of carbon frameworks in Li–S batteries has been demonstrated to be promising, challenges still remain in the practical production of such materials using inexpensive and non-toxic ingredients. This has stimulated a need to develop new, alternative cathode frameworks, examples of which include metal−organic frameworks^36^,^37^,^38^ and conducting polymer shells^39^,^40^,^41^,^42^. However, to date, better battery performance from these materials, as compared with that from structured carbon materials, has only rarely been reported.

In recent reports, Pyun and coworkers^43^,^44^,^45^ have proposed a noteworthy strategy to develop high-performance Li–S batteries by synthesizing sulfur-containing polymers directly from elemental sulfur in large quantities, at low cost. The specific capacity of Li–S cells fabricated using these polymers as the cathode active materials was as high as 1,005 mAh g^−1^ at 100 cycles, 817 mAh g^−1^ at 300 cycles and 635 mAh g^−1^ at 500 cycles (at 0.1 C). These are undoubtedly promising results for future Li–S battery technologies, but the poor conducting nature of sulfur-containing polymers appears to be a fundamental impediment to achieving rate cycling performance, as revealed by a rapid reduction in the specific capacity to <400 mAh g^−1^ with an increase in the cycling rate to 2 C (refs 44, 45). In fact, the high-rate performance of any LIBs based on organic electrode materials has long been a substantial challenge that is of importance for fast charging energy storage systems.

Here we report the development of a new Li–S battery composed of sulfur-containing polymers as a cathode active material that demonstrates high specific capacities, lifetimes up to 450 cycles with excellent capacity retention over 83% and notable rate capability at various current rates from C/10 (1,210 mAh g^−1^) to 5 C (730 mAh g^−1^). To our knowledge, this is the best performance of any battery using organosulfur cathode materials reported to date. The key to this success is a facile synthesis of organosulfur compounds with controllable morphology from elemental sulfur using porous trithiocyanuric acid (TTCA) crystals as a soft template. This is a unique feature of our systems relative to other vulcanized polymers reported in the literature, where the controls of shape and morphology were not practical. In particular, the amine groups of TTCA were found to facilitate fast Li^+^-ion transport within cathode frameworks during battery cycling, and are intimately associated with the improved rate capability of these Li–S cells. The size- and shape-controlled soft-template synthesis of sulfur-containing polymers sets new trends and provides ideas for avenues of further research to advance Li–S battery technologies.

## Results

### A soft-template synthesis for sulfur-rich polymers

**Figure 1:**
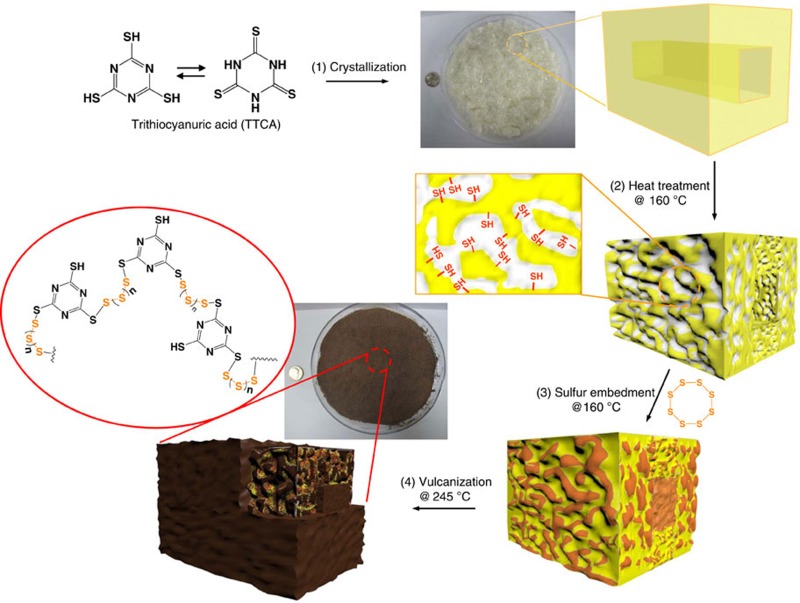
Synthetic route of sulfur-rich polymers in tens of grams. Schematic drawings describing the synthetic procedures of sulfur-rich polymers with controllable morphology; (1) preparation of TTCA co-crystals, (2) creation of porous TTCA frameworks by removing the solvents via heat treatment, (3) impregnation of sulfur into porous TTCA frameworks and (4) ring-opening polymerization of elemental sulfur at the thiol surfaces of porous TTCA template, following the equation given in the figure.

We employed porous TTCA crystals as a soft template for the synthesis of sulfur-containing polymers. Figure 1 provides a schematic description of the synthetic procedures used for sulfur-rich polymers with controllable morphology in tens of grams, which can be summarized in the following steps: (1) preparation of a set of TTCA co-crystals by varying the crystallization solvents, formed by N−H···S hydrogen bonds between TTCA molecules and N−H···O=C hydrogen bonds between TTCA and the solvents, (2) creation of porous TTCA frameworks by removing the solvents via simple heat treatment, (3) impregnation of sulfur into porous TTCA frameworks and (4) ring-opening polymerization of elemental sulfur at the thiol surfaces of porous TTCA frameworks, following the equation given in the figure. Detailed procedures are provided in the Methods.

**Figure 2:**
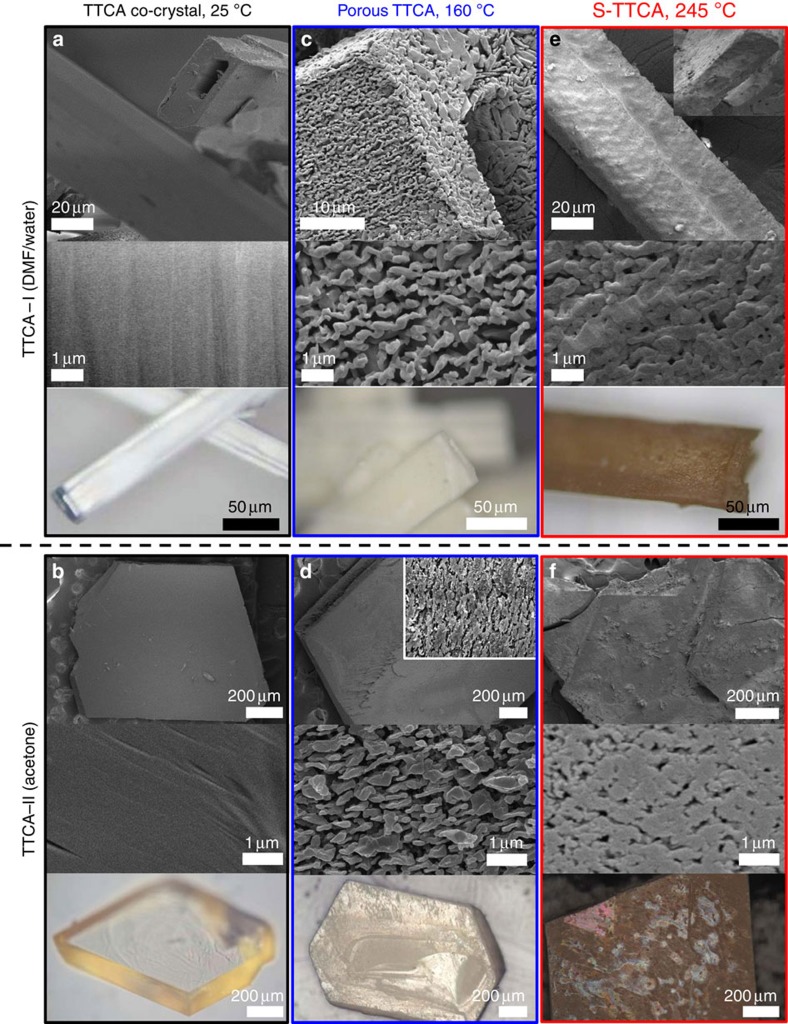
Vulcanized TTCAs having different morphologies. (**a**,**b**) SEM and OM images of TTCA-I and TTCA-II co-crystals prepared by varying crystallization solvents, described as rectangular tubes and sliced plates, respectively. (**c**,**d**) SEM and OM images of the TTCA-I and TTCA-II after the removal of solvents at 160 °C, presenting the appearance of interconnected polydisperse pores. (**e**,**f**) SEM and OM images of the S-TTCA-I and S-TTCA-II synthesized at 245 °C using the porous TTCAs as a soft template. The SEM images confirm the restoration of smooth surfaces and disappearance of most of the pores after the vulcanization.

By using two different solvents, that is, dimethylformamide (DMF)/water (1:1 vol) co-solvent and acetone, two types of TTCA co-crystals with different morphologies, rectangular tubes and splice plates, respectively, were obtained. Hereafter, the co-crystals are referred to as TTCA-I (DMF/water) and TTCA-II (acetone). As examined by scanning electron microscopy (SEM) and optical microscopy (OM), the TTCA-I crystals are ∼50 × 20 μm and hundreds of microns in length, and have a rectangular hole (Fig. 2a), while the TTCA-II crystals are ∼1 × 1 mm rhombi with a thickness of 150 μm (Fig. 2b). Removal of solvent from TTCA-I and TTCA-II at 160 °C resulted in the appearance of intriguing rough surfaces, as shown in Fig. 2c,d, which include the formation of interconnected polydisperse pores in the range of a few tens of nanometres to a few microns throughout the crystals. The evolution of porous morphologies can be readily perceived from the changes in transparency of the crystals, as can be seen from OM images. The amounts of solvents in TTCA-I and TTCA-II were determined by thermogravimetric analysis (TGA) to be 30 wt% and 14 wt%, respectively (Supplementary Fig. 1), implying that the surface area of the heat-treated TTCA-I and TTCA-II are fundamentally different. Nevertheless, both crystals are still described as having the same morphologies, including the unperturbed rectangular hole of TTCA-I.

Sulfur-containing polymers were synthesized using the porous TTCA crystals as a soft-template. A two-step vulcanization process was carried out in a sealed vessel—a low-temperature step at 160 °C to embed sulfur into the porous TTCA frameworks, followed by further heating to 245 °C to cause ring-opening polymerization of elemental sulfur (S_8_) into a linear polysulfane along the thiol surfaces. Hereafter, the vulcanized TTCAs are denoted as S-TTCA-I and S-TTCA-II. This process was accompanied by a colour change of the crystals from pale yellow to dark brown. Since S-TTCA-I and S-TTCA-II are insoluble in any solvent, it is inferred that the diradical ends of polysulfane form crosslinks between TTCA molecules. The SEM images in Fig. 2e,f confirm the restoration of smooth surfaces and disappearance of most of the pores after the vulcanization. This includes S-TTCA-I that is devoid of tubular holes after the reaction, as clearly shown in the inset image.

### Molecular and structural characterization of vulcanized TTCAs

**Figure 3:**
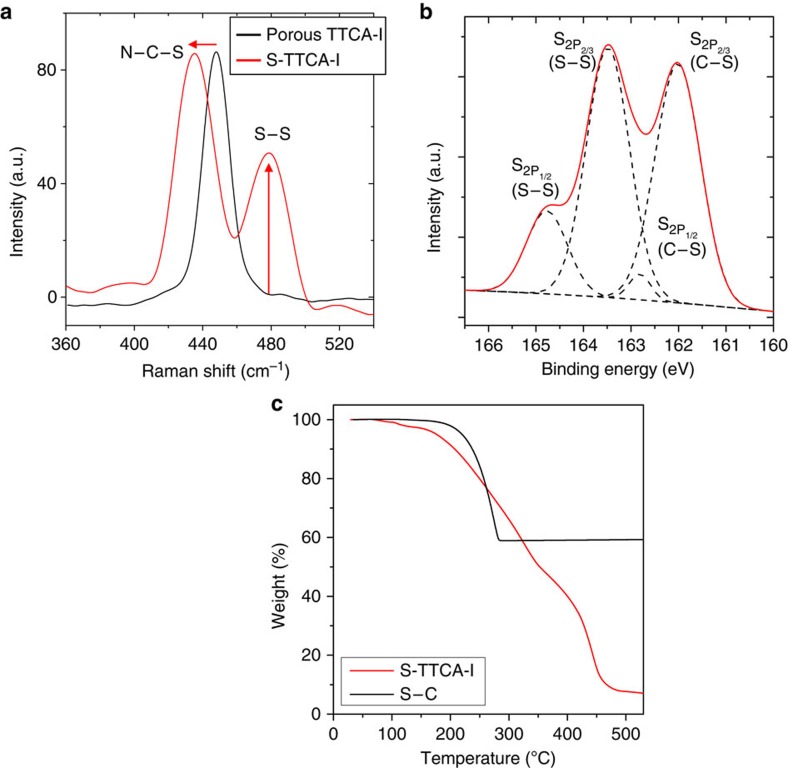
Molecular characteristics of vulcanized TTCAs. (**a**) Raman spectra of porous TTCA-I (heat treated at 160 °C) and S-TTCA-I (vulcanized at 245 °C), (**b**) XPS profile of the S-TTCA-I and (**c**) TGA analysis of the S-TTCA-I, compared with that of elemental sulfur in carbon frameworks (S–C).

Molecular characteristics of the vulcanized TTCAs were examined by Raman spectroscopy. Representative spectra of porous TTCA-I and S-TTCA-I are shown in Fig. 3a. The peak centered at 448 cm^−l^ was attributed to N–C–S deformation and was evident in porous TTCA-I. After vulcanization, the characteristic N–C–S deformation peak was shifted to 435 cm^−l^ and was accompanied by the appearance of new peak at 482 cm^−l^ as evidence for the formation of the S–S bonds. This indicates that elemental sulfur (S_8_) reacts with the thiol groups of TTCA crystals, following the equation in Fig. 1.

By combining X-ray photoelectron spectroscopy (XPS) and TGA experiments, the chemical states and sulfur contents in the vulcanized TTCAs were further investigated. The representative XPS profile of S-TTCA-I, shown in Fig. 3b, indicates that ∼60% of the sulfur is present in the form of the S–S bonds, while 40% exists in the form of C–S bonds. The total sulfur content in S-TTCA-I was determined to be 63 wt% by TGA analysis (Fig. 3c), which is not much different in S-TTCA-II (58 wt%, and thus the data are not shown here). Since the vulcanized TTCAs are insoluble in any solvent, the molecular structure of S-TTCA-I was further investigated by monitoring the quenched reaction intermediates using the positive-ion electrospray ionization time-of-flight mass spectrometry. As shown in Supplementary Fig. 2, the molar ratio of TTCA and sulfur was determined to be 1:7 for the major product and thus, the averaged *n* value in the –S_n_– chains (Fig. 1) for the S-TTCA-I is inferred to be 7.

Notably, as shown in Fig. 3c, the weight loss of sulfur in S-TTCA-I starts at 110 °C and continues to 310 °C. This is considerably different from the evaporation of sulfur in a carbon framework (S–C, 40 wt% of sulfur), where weight loss begins at around 180 °C, and is complete by 280 °C. The low sublimation temperature and broad decomposition temperature window of sulfur in S-TTCA-I indicate that small sulfur molecules are covalently bound to the TTCA frameworks. Weight loss in TTCA begins at 360 °C and is complete by 500 °C.

**Figure 4:**
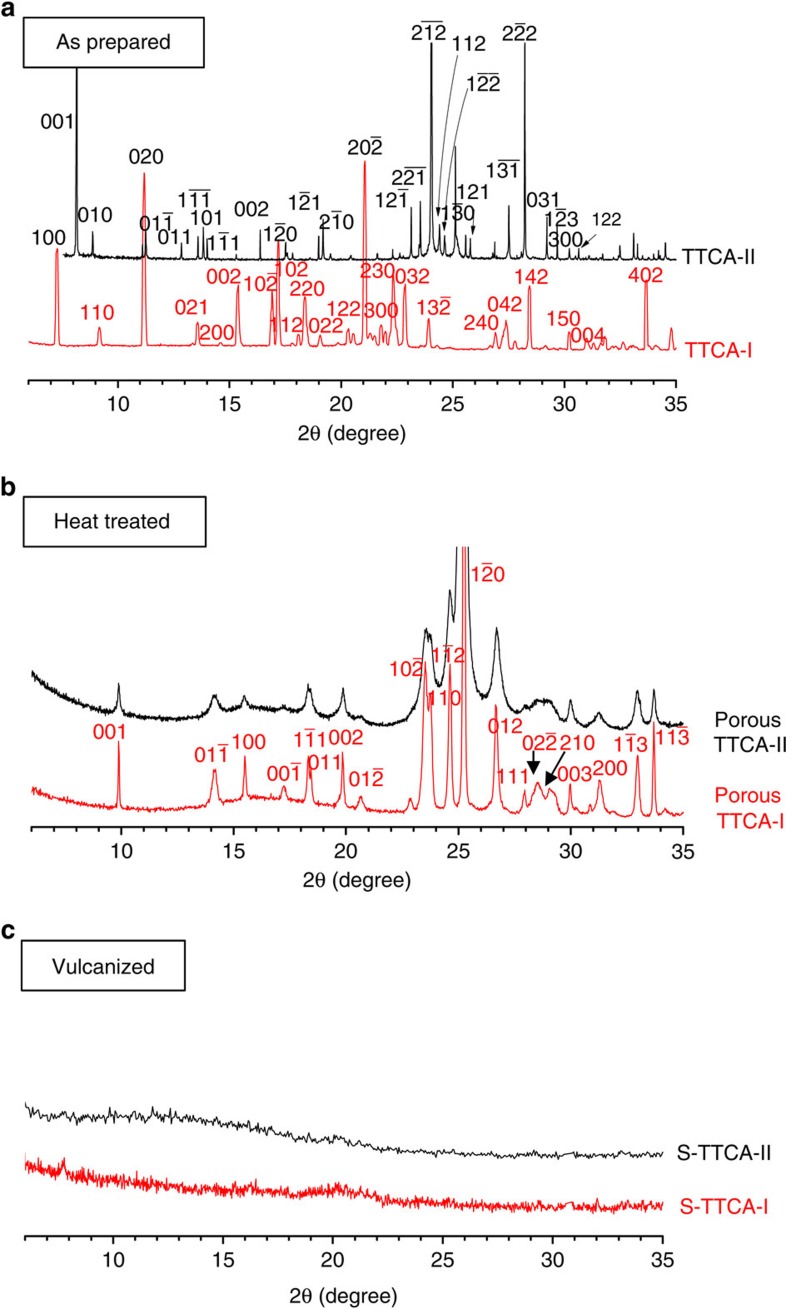
Crystal structure analysis. Powder XRD profiles of (**a**) the as-prepared TTCA-I and TTCA-II co-crystals, (**b**) the heat-treated TTCA-I and TTCA-II at 160 °C and (**c**) the vulcanized S-TTCA-I and S-TTCA-II at 245 °C. Miller indices of the reflection plane *hkl* are given in each figure.

To determine the changes in crystal structures as a result of heat treatment and vulcanization, a set of powder X-ray diffraction (XRD) profiles that used a 2*θ* scan range of 5−35° with a 0.02° step interval are presented in Fig. 4a–c. As shown in Fig. 4a, TTCA-I initially had a monoclinic P2_1/c_ space group, while a triclinic P1 space group was determined for TTCA-II. The unit cell parameters obtained from the Cambridge Structural Database are *a*=9.780 Å, *b*=12.755 Å, *c*=9.280 Å, *α*= 90.00°, *β*=91.19° and *γ*=90.00° for TTCA-I; and *a*=8.937 Å, *b*=9.985 Å, *c*=10.447 Å, *α*=95.12°, *β*=96.79° and *γ*=107.29° for TTCA-II^46^. Elimination of solvent from TTCA-I and TTCA-II then leads to structural transformation into identical triclinic structures with a 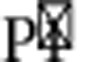 space group (Fig. 4b) having the unit cell parameters *a*=5.587 Å, *b*=7.047 Å, *c*=8.799 Å, *α*=102.99°, *β*=92.87° and *γ*=110.47°. The emergence of porous morphologies is thus ascribed to shrinkage of the cell volume, where the degree of reduction for TTCA-I and TTCA-II is different. This should be closely related with the surface area (pore volume) of heat-treated TTCA frameworks. Finally, S-TTCA-I and S-TTCA-II displayed featureless XRD patterns, as shown in Fig. 4c, denoting that the covalent attachment of sulfur into TTCA frameworks destroyed the long-ranged π–π stacking of TTCA rings. Given that the crystalline peaks of both TTCA and elemental sulfur remain intact after sulfur impregnation at 160 °C (Supplementary Fig. 3), the absence of sulfur crystal peaks after vulcanization (Fig. 4c) should be noteworthy. This implies that the sulfur in S-TTCA exists in an amorphous state, as it was involved in the polymerization. Note that the TTCA crystals were thermally stable (see the temperature-dependent XRD profiles in Supplementary Fig. 4), and therefore, the loss of crystallinity of S-TTCA is not ascribed to the amorphization of TTCA.

### Battery performance of the Li-S cells

**Figure 5:**
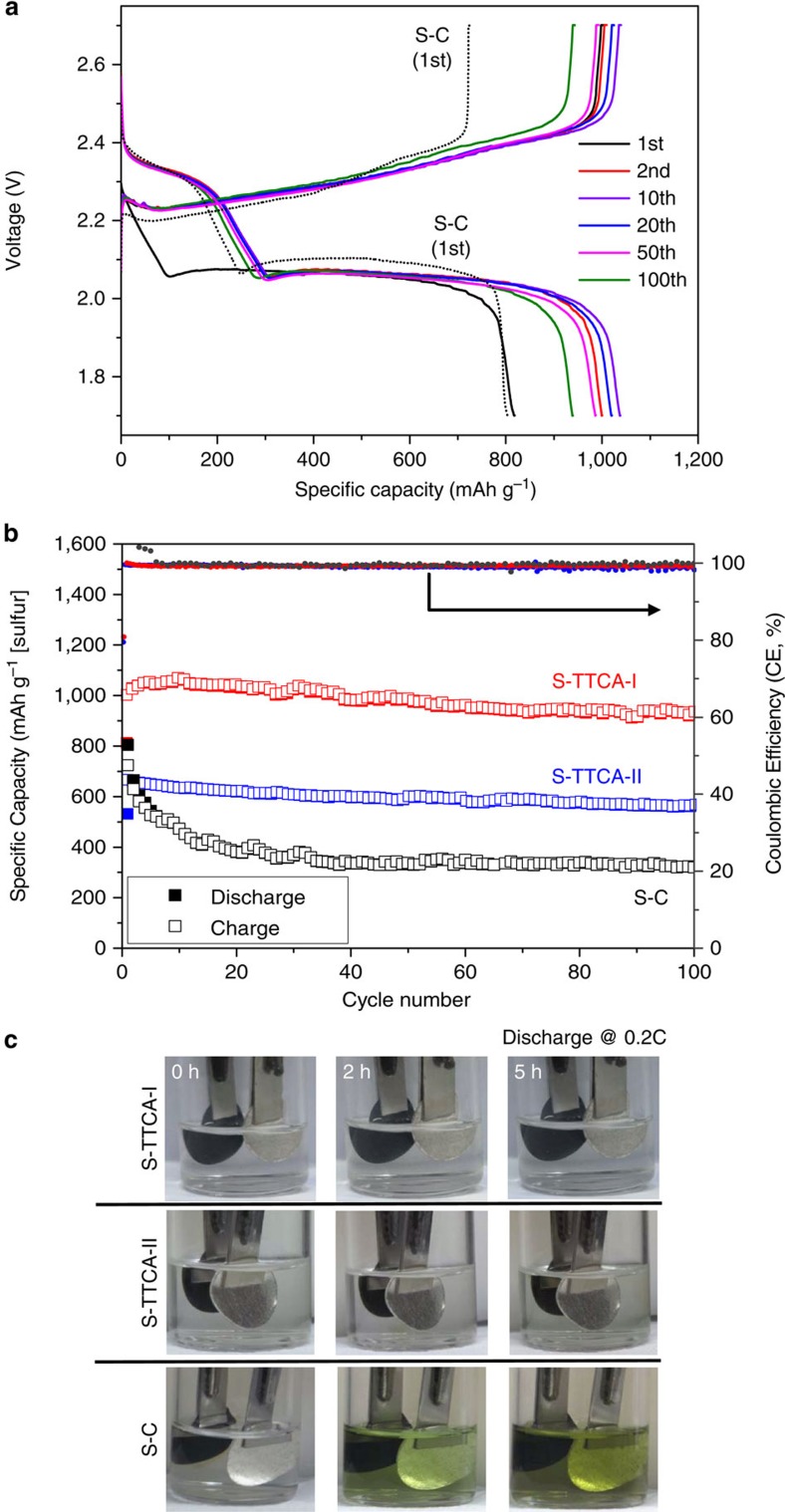
Battery performance of Li–S cells. (**a**) Representative galvanostatic discharge/charge voltage profiles of the Li/S-TTCA-I cell, cycled between 1.7 and 2.7 V at 0.2 C at room temperature. The discharge/charge voltage profiles of the Li/S-C cell obtained at the first cycle are also shown as dashed lines. (**b**) The discharge/charge capacities and Coulombic efficiencies of the Li/S-TTCA-I and Li/S-TTCA-II cells, compared with those of conventional Li/S–C cells. (**c**) Photographs displaying the dissolution of polysulfide intermediates into electrolyte during discharge at 0.2 C for the Li/S–C beaker cell, contrary to the colourless electrolytes of the Li/S-TTCA cells.

Sulfur cathodes were fabricated by integrating S-TTCA-I (or S-TTCA-II), Super P carbon and polyvinylidene (PVDF) binder. Conventional sulfur cathodes composed of elemental sulfur, Super P carbon and PVDF (40 wt% of sulfur) were used as controls. After assembling coin cells containing a Li-metal anode, liquid electrolyte and the sulfur cathode, discharge/charge cycle properties of the cells at room temperature were examined. Figure 5a shows representative galvanostatic discharge/charge voltage profiles of the Li/S-TTCA-I cell, cycled between 1.7 and 2.7 V at 0.2 C (1 C=1,675 mA g^−1^). Only one distinct plateau at 2.06 V (vs. Li/Li^+^) was seen during the first discharge process for the Li/S-TTCA-I cell, in contrast to two plateaus for the Li/S–C cell at 2.35 and 2.10 V (dashed lines). This denotes that most of the sulfur in the S-TTCA-I electrode is bound to TTCA frameworks by forming disulfide bonds. After the first discharge/charge cycle, two stable discharge plateaus appeared at 2.33 and 2.06 V, ascribed to the appearance of S_8_ after electrochemical scission and regeneration of disulfide bonds with cycling. Overall, the discharge/charge voltage profiles of the Li/S-TTCA-II cell are similar to those of the Li/S-TTCA-I cell in their absence of the ring-opening plateau at 2.33 V during the first discharge cycle (see Supplementary Fig. 5).

Figure 5b presents the discharge/charge capacities (based on sulfur weight) of the Li/S-TTCA-I, Li/S-TTCA-II and Li/S–C cells at 0.2 C. The Li/S-TTCA-I cell shows a first discharge capacity of 813 mAh g^−1^, which stabilized around 1,050 mAh g^−1^ after five cycles. A high capacity of 945 mAh g^−1^ was maintained after 100 cycles, corresponding to 92% capacity retention compared with the second discharge capacity, with a high coulombic efficiency of >99% throughout. It is thus inferred that polysulfide intermediates confined within S-TTCA-I remain impermeable during cycling. These results are in sharp contrast to the low discharge capacity and poor capacity retention seen in the Li/S–C cell (323 mAh g^−1^, 40%) after 100 cycles, ascribed to the well-known polysulfide shuttle effects. The Li/S-TTCA-II cell also exhibited a good capacity retention of 86% (compared with the second discharge capacity) and a high coulombic efficiency of 99%, except for considerably lower specific capacities (565 mAh g^−1^ after 100 cycles) than for the Li/S-TTCA-I cell.

We note that the battery performance of porous TTCA/S composite cathodes (sulfur was impregnated, but no vulcanization reaction was processed) was distinctly different from that of S-TTCA cathodes, as rapid capacity loss incurred for the composite cathodes during the initial 20 cycles (Supplementary Fig. 6). This clearly indicates the role of covalent attachment of sulfur to the porous TTCA frameworks to improve cycling performance. We also note that there are slight differences in discharge plateaus for S-TTCA (2.06 V) and TTCA/S composite (2.10 V), where that of S–C is 2.10 V.

To further underpin the improved capacity retention properties of S-TTCA cathodes, compared with conventional S–C cathodes, we carried out beaker cell experiments. Representative results are given in Fig. 5c. The increasingly dark green–yellow colour of electrolyte was observed for the Li/S–C cell during discharge, contrary to the colourless electrolytes for the Li/S-TTCA cells.

**Figure 6:**
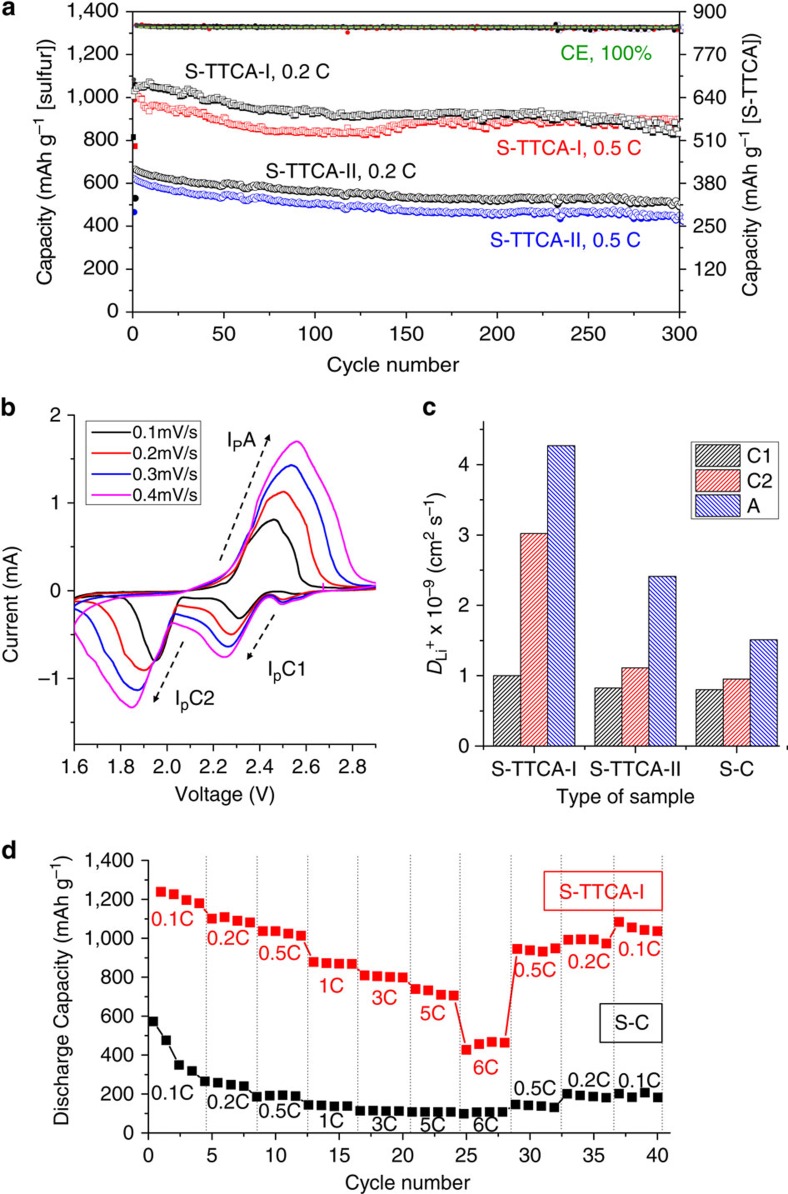
High-capacity and high-rate Li–S battery. (**a**) The discharge/charge capacities and Coulombic efficiencies of the Li/S-TTCA-I and Li/S-TTCA-II cells for 300 cycles at different C rates. (**b**) Representative voltammograms of the S-TTCA-I cathode obtained at different scan rates and (**c**) Li^+^-ion diffusion coefficients of the S-TTCA-I, S-TTCA-II and S–C cathodes, calculated using the Randles–Sevcik equation. (**d**) Rate performance of the Li/S-TTCA-I cell, compared with that of the Li/S–C cell.

The better battery performance with the S-TTCA-I cathode rather than with the S-TTCA-II cathode was repeatedly observed at different C rates with an extended life of 300 cycles. Discharge–charge capacities of the Li/S-TTCA-I and Li/S-TTCA-II cells at 0.2 and 0.5 C are shown in Fig. 6a. The Li/S-TTCA-I cell can deliver 872 mAh g^−1^ (at 0.2 C) and 886 mAh g^−1^ (at 0.5 C) after 300 cycles, with excellent capacity retention of over 85%. In contrast, low discharge capacities of 513 mAh g^−1^ (at 0.2 C) and 440 mAh g^−1^ (at 0.5 C) were attained with the S-TTCA-II cathode after 300 cycles. This clearly signals the morphological advantages of S-TTCA-I in enhancing the performance of the Li–S cells.

## Discussion

The markedly improved battery performance of the Li/S-TTCA-I cell, compared with the Li/S-TTCA-II and Li/S-C cells, was found to be closely associated with Li^+^-ion transport in the sulfur cathodes. Cyclic voltammetry analysis of the S-TTCA-I, S-TTCA-II and S–C cathodes was carried out to evaluate the Li^+^-ion diffusion coefficients (*D*_Li_) of the cathodes using the Randles–Sevcik equation, as described below,


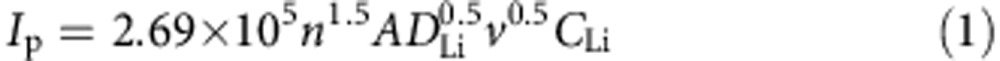


where *I*_p_ indicates the peak current, *n* is the number of electron in the reaction, *A* is the electrode area, *v* is the scanning rate and *C*_Li_ is the lithium-ion concentration in the electrolyte. Representative voltammograms obtained with the S-TTCA-I cathode are shown in Fig. 6b.

From the linear relationship of *I*_p_ and *v*^0.5^, 
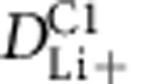
 (hereafter C1, cathodic peak at ∼2.3 V), 
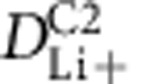
 (C2, cathodic peak at ∼ 1.9 V) and 
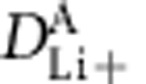
 (A, anodic peak at ∼2.4 V) were obtained. For the S-TTCA-I, cathode C1=1.0 × 10^−9^, C2=3.0 × 10^−9^ and A=4.3 × 10^−9^ cm^2^ s^−1^ were determined, for which the values were up to 2.7 times larger than those of the S-TTCA-II cathode (C1=8.2 × 10^−10^, C2=1.1 × 10^−9^ and A=2.4 × 10^−9^ cm^2^ s^−1^), as plotted in Fig. 6c. This suggests that the TTCA-I tubular framework morphology allows easy access of liquid electrolytes to active materials through three-dimensionally interconnected hierarchical pores, leading to facilitated fast Li^+^-ion transport. Conversely, expeditious Li^+^-ion migration in two-dimensional S-TTCA-II with a thick wall of 150 μm (see, for example, wall thickness of TTCA-I was 20 μm Fig. 1) seemed to be impaired. The lowest *D*_Li+_ values of the S–C cathode, C1=8.0 × 10^−10^, C2=9.5 × 10^−10^ and A=1.5 × 10^−9^ cm^2^ s^−1^ also lead us to conclude that the organized Li^+^-coordination sites (secondary amine groups and π electrons) of the TTCA rings raised the Li^+^-ion transfer rate.

By virtue of fast Li^+^-ion transport in the S-TTCA-I cathode, noticeable rate capability was demonstrated for the Li/S-TTCA-I cell. As shown in Fig. 6d, the cell was found to deliver a reversible capacity of 1,210 mAh g^−1^ (0.1 C), 1,090 mAh g^−1^ (0.2 C), 1,030 mAh g^−1^ (0.5 C), 872 mAh g^−1^ (1 C), 803 mAh g^−1^ (3 C) and 730 mAh g^−1^ (5 C). Although a further increase in the current value to 6 C resulted in substantial reduction in the discharge capacity to 452 mAh g^−1^, further cycling at low rates (after cycling at various rates) brought the cell back to a reversible capacity of 1,053 mAh g^−1^ at 0.1 C. To the best of our knowledge, our results represent the best rate performance in any existing report on organosulfur cathodes. Control experiments using the S–C cathode are also shown in Fig. 6d, which indicate greatly decreased capacity values under 1C (125 mAh g^−1^), 3 C (117 mAh g^−1^) and 5 C (102 mAh g^−1^), analogous to most organosulfur cathodes.

It should be noted that the covalent attachment of sulfur to TTCA resulted in a large decrease in bandgap of the TTCA molecule. We carried out quantitative investigation on the electronic structures of TTCA and S-TTCA by *ab initio* calculations at 0 K in a vacuum using a density functional theory exchange-correlation functional. As shown in Supplementary Fig. 7, for the neat TTCA, the bandgap of 5.76 eV was determined at the B3PW91/6-31G** level, which decreased to 4.59 eV as a result of S_6_ attachment (the shorter S_*n*_ yielded larger bandgap, that is, 4.84 eV of TTCA-S_4_). Considering the fact that our S-TTCA is a polymeric system, many experimental parameters such as polydispersity in chain length and compositional fluctuations will lead to additional reduction in the bandgap, enabling the exceptional high-rate performance of our Li/S-TTCA cells.

Currently, the Li/S-TTCA-I cell has been extended to 450 cycles, while retaining a significant capacity of 850 mAh g^−1^ (capacity retention of 83%) with high coulombic efficiency of 99% throughout (Supplementary Fig. 8). Experiments on whether the cathode loading effects are present in the battery performance^47^ will be a subject of our future studies. Our preliminary examination indicates analogous battery performance with a twofold increase in cathode loading. Further improvement of the specific capacity by optimizing the liquid electrolyte and type of polymeric binder is currently underway.

In summary, we explored a new methodology to improve the performance of Li–S batteries based on organosulfur cathodes. Using porous organic crystal templates, tens of grams of sulfur-rich polymers with different morphologies were synthesized from elemental sulfur. This is a unique feature of our system relative to other vulcanized polymers reported in the literature, where control of shape and morphology were not viable. The successful implementation of three-dimensionally interconnected sulfur-rich phases in the cathode frameworks enabled us to achieve high specific capacities of 850 mAh g^−1^ after 450 cycles with an unprecedented capacity retention of over 83%, attributed to impermeable polysulfide intermediates confined within the vulcanized polymers during cycling. Our work demonstrated one of the highest specific capacities reported for Li–S batteries, particularly at high current rates, that is, 1 C (872 mAh g^−1^), 3 C (803 mAh g^−1^) and 5 C (730 mAh g^−1^). This is ascribed to the organized Li^+^-ion coordination sites of organic crystals, allowing the seamless transport of Li^+^-ion into active materials. We also gained a better understanding of the factors affecting battery performance—organic crystal morphology appears to play a central role in determining the specific capacity by providing fundamentally different surface areas, thereby affecting and Li^+^-ion transport rate.

## Methods

### Preparation of TTCA co-crystals

TTCA-I and TTCA-II co-crystals were prepared by varying crystallization solvent. For the TTCA-I, TTCA (98%, TCI) was dissolved in 1:1 co-solvent of deionized water and dimethylformamide (DMF, 99%, Alfa Aesar), followed by crystal growth at 0 °C for 24 h. The resultant crystals were collected by filtration and vacuum dried at 40 °C for 24 h to remove any residual solvents. The TTCA-II co-crystals were prepared by dissolving the TTCA in acetone (HPLC grade, J. T. Baker), followed by slow solvent-evaporation at 50 °C. Both TTCA-I and TTCA-II co-crystals revealed a clear yellow colour.

### Synthesis of S-TTCA-I and S-TTCA-II

S-TTCA-I and S-TTCA-II were synthesized directly from elemental sulfur using the TTCA-I and TTCA-II crystals as a soft template. A three-step process was applied: (1) The TTCA-I and TTCA-II were exposed to *T*=160 °C to remove the solvents in the crystals. The removal of solvents from TTCA-I and TTCA-II co-crystals resulted in the changes in transparency of the crystals. (2) On the completion of the solvent removal, predetermined amounts of sulfur (at 1:3 weight ratio of TTCA:sulfur) are embedded into the TTCA frameworks at 160 °C for 10 h under an argon atmosphere. (3) The mixture was further heated at 245 °C for 2 h to stimulate ring-opening polymerization of elemental sulfur (S_8_) into a linear polysulfane along the thiol surfaces of TTCA frameworks. The resultant S-TTCA-I and S-TTCA-II showed a dark brown colour.

### Morphology and structure characterization

Morphologies of the TTCA crystals before/after heat treatment and vulcanization were determined by combining optical microscope (Zeiss axio scope, A1) and field emission scanning electron microscope (XL30S FEG, Philips). Powder XRD analysis on each crystal was carried out at 9B HRPD beamline (*λ*=1.4640 Å) of Pohang Accelerator Light Source.

### Fourier transform Raman experiments

Confocal Raman spectra were measured using a WITEC Alpha 300R Raman spectroscope (WITec, Ulm, Germany), equipped with a HeNe laser. The spatial resolution of the spectrometer was 250 nm. Laser excitation power was adjusted below 3 mW to reduce potential thermal damage caused by the laser source.

### Molecular Characterization of S-TTCA

XPS experiments were conducted by using an Escalab 250xi spectrometer employing a monochromatic Al-Kα X-ray source and hemispherical electrostatic analyser. For characterizing the sulfur weights in the S-TTCA-I and S-TTCA-II, TGA was performed in a temperature range of 25–550 °C with a heating rate of 10 °C min^−1^ under a nitrogen atmosphere. The positive-ion electrospray ionization time-of-flight (Compact, Bruker) mass spectra of quenched products were acquired. The mass spectrometer was operated with a source temperature of 180 °C and a capillary voltage of 4,500 V. The *m/z* range of the mass spectrometer was 50–1,700 Da.

### Preparation of sulfur cathodes

For the S-TTCA-I and S-TTCA-II cathodes, 60 wt% of S-TTCA-I (or S-TTCA-II), 30 wt% of Super P carbon (Alfa Aesar) and 10 wt% of PVDF (Solef) binder were dispersed in NMP (Sigma Aldrich) by ball milling (MillMM400) under an argon atmosphere. The ball-milled slurry was casted onto Al-foil current collector by a doctor-blade method. The total cathode loading was 2.0 mg cm^−2^. The amount of S-TTCA in the cathode was 1.2 mg cm^−2^, of which sulfur content was 0.8 mg cm^−2^. Conventional sulfur cathodes were also prepared according to the same procedures with sulfur powder (99.998%, Sigma Aldrich), Super P carbon and PVDF binder in weight ratio of 40:50:10. All three sulfur cathodes were dried at 50 °C for 24 h under Ar-blanket, followed by vacuum drying at 50 °C for 24 h. The dried electrodes were cut by a disc cutter (MTI, 15 mm diameter).

### Battery tests

The Li–S cells were assembled in a high-purity Ar-filled glove box to avoid any possible contamination by moisture and oxygen. Coin type (CR2032, MTI) cells were fabricated by assembling a Li-metal anode, a porous polypropylene separator (Celgard 2400) and sulfur cathode. The liquid electrolyte was prepared by dissolving 1 M lithium bis (trifluoromethane)sulfonamide (LiTFSI, 98.0%, TCI) and 0.2 M lithium nitrate (99.999%, Alfa Aesar) in a mixture of tetraethylene glycol dimethyl ether (TEGDME, Sigma Aldrich) and 1,3-dioxolane (DIOX, 99.8%, Sigma Aldrich; 33:67 vol%). Galvanostatic discharge/charge tests on the Li–S cells were performed by cycling between 1.7 and 2.7 V at predetermined current rates using battery cycler (WBCS3000, Wonatech). The cyclic voltammetry analysis on the sulfur cathodes were performed in the potential range of 1.7–2.7 V by varying the scan rate.

## Additional information

**How to cite this article:** Kim, H. *et al*. Synthesis of three-dimensionally interconnected sulfur-rich polymers for cathode materials of high-rate lithium–sulfur batteries. *Nat. Commun.* 6:7278 doi: 10.1038/ncomms8278 (2015).

## Supplementary Material

Supplementary Information Supplementary Figures 1-8
