# Supplementary material for: Synthesis of three-dimensionally interconnected sulfur-rich polymers for cathode materials of high-rate lithium–sulfur batteries
Source: Nat Commun. 2015 Jun 12;6:7278. doi: 10.1038/ncomms8278 (PMC4490390; doi:10.1038/ncomms8278)
Supplement: Supplementary Information — Supplementary Figures 1-8 [file ncomms8278-s1.pdf]

Supplementary Figures

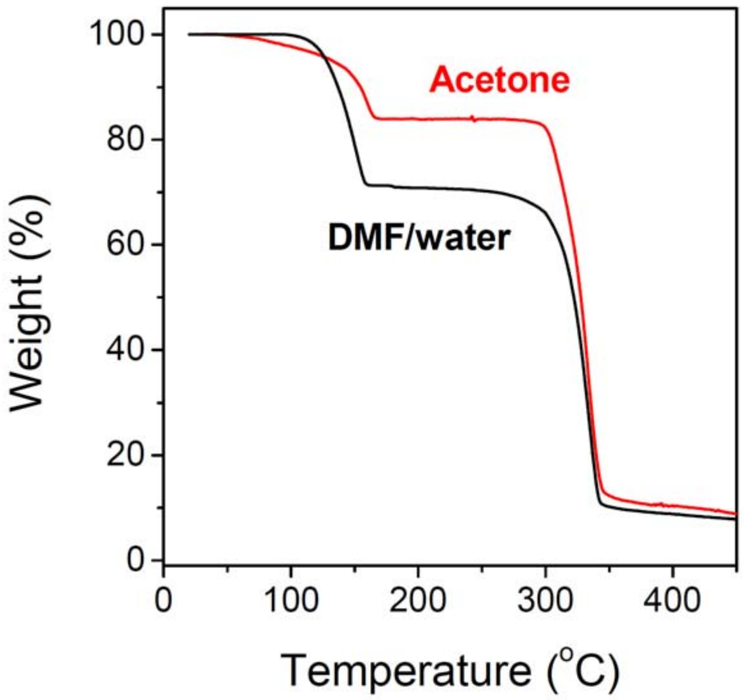

Supplementary Fig. 1. The amounts of solvents in TTCA-I and TTCA-II. Thermogravimetric analysis (TGA) data of TTCA-I (DMF/water) and TTCA-II (acetone) co-crystals. TGA runs were conducted at a constant heating rate of 10 ^{o}C/min under a nitrogen atmosphere. From the weight loss curves, the amounts of solvents in TTCA-I and TTCA-II were determined to be 30wt% and 14wt%, respectively.

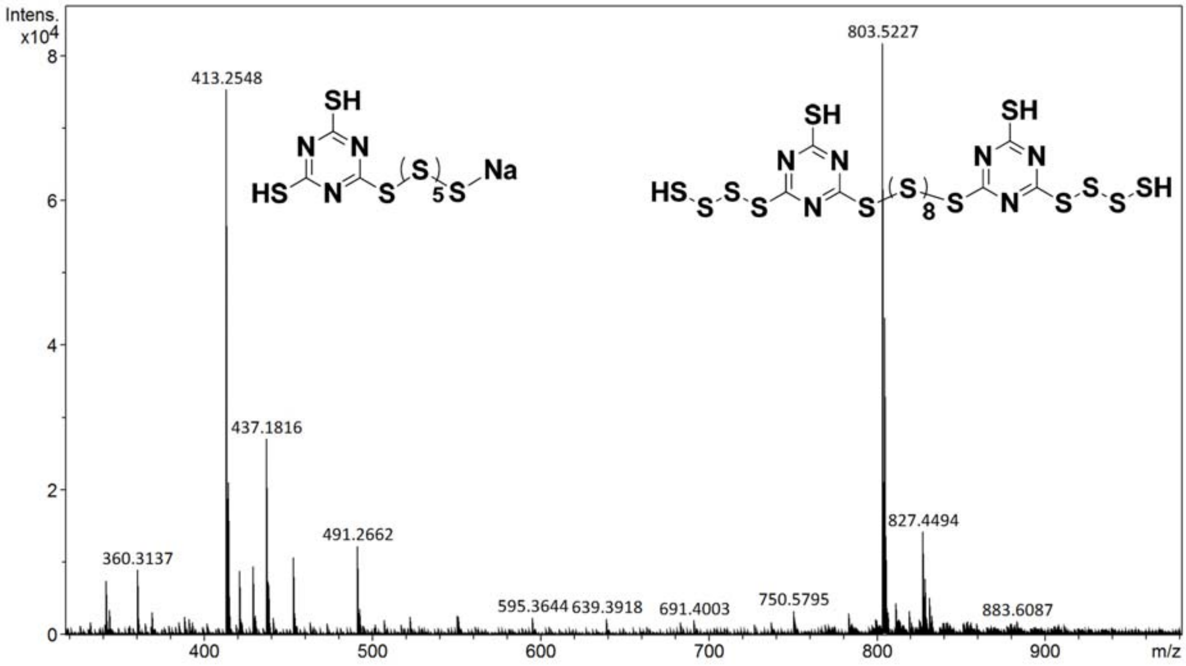

Supplementary Fig. 2. The molar ratio of TTCA and sulfur in vulcanized products. The positive-ion electrospray ionization time-of-flight (ESI-TOF) mass spectrum of the quenched reaction intermediates of S-TTCA-I during vulcanization reaction, suggesting the molar ratio of TTCA and sulfur as 1:7 for the major product. Expected molecular structures of the reaction intermediates are given in the spectrum. After the completion of vulcanization reaction, the vulcanized products are not soluble in any organic solvents

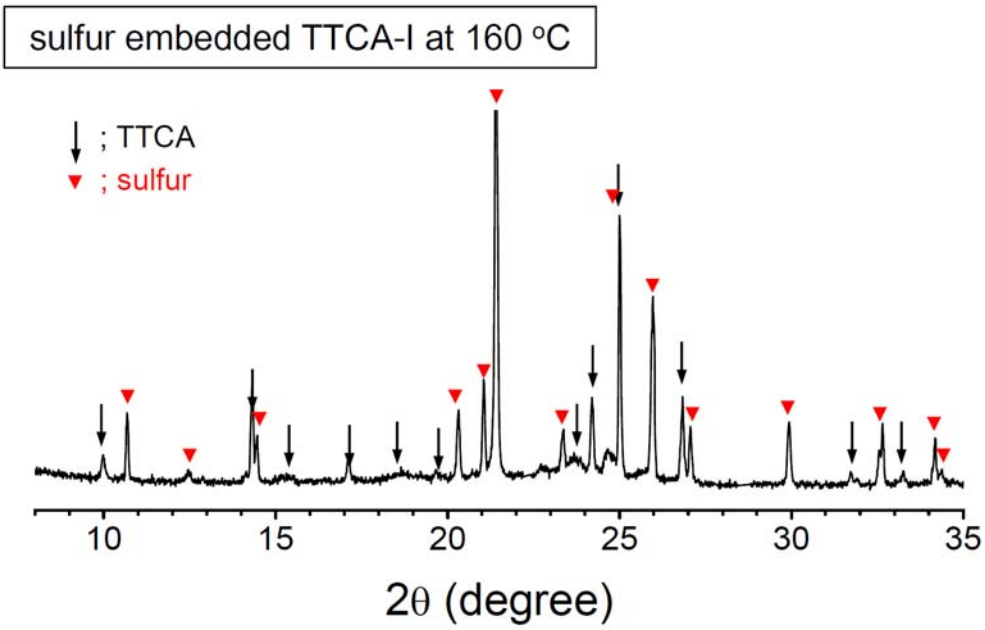

Supplementary Fig. 3. Crystalline characteristics of sulfur after impregnating in TTCA frameworks. Power X-ray diffraction (XRD) profile of sulfur impregnated TTCA-I frameworks at low temperature step of 160 ^{o}C. Crystalline peaks of both TTCA and elemental sulfur remain intact, as indexed in the figure. This implies that the sulfur exists in crystalline S_8 state at 160 ^{o}C, which transformed into amorphous state at 245 ^{o}C (Figure 4c), as it involved in the polymerization.

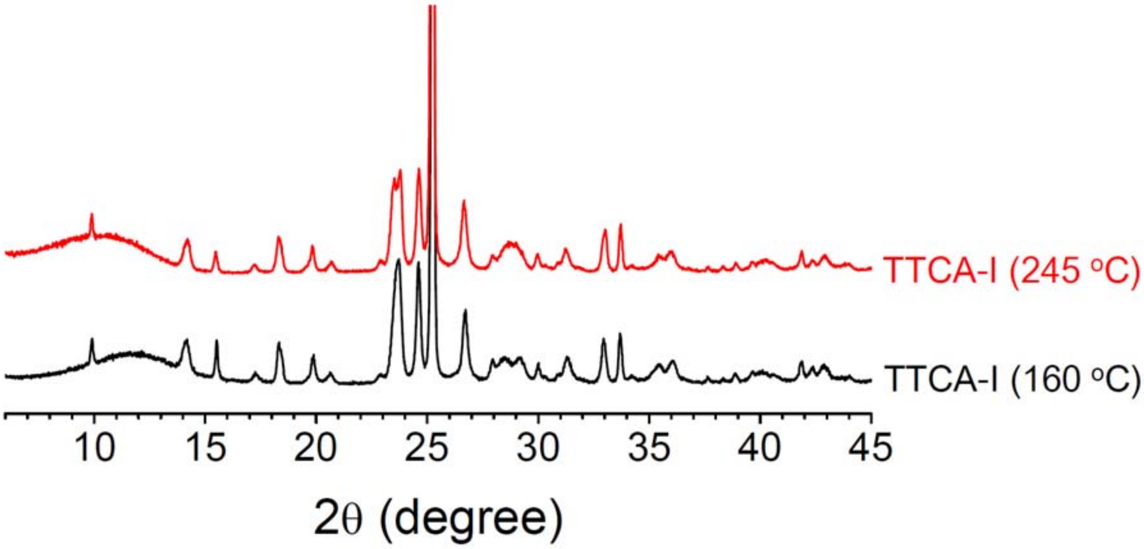

Supplementary Fig. 4. Thermal stability of TTCA crystals. Powder X-ray diffraction (XRD) profiles of the heat-treated TTCA-I crystals, measured at 160 ^{o}C and 245 ^{o}C, where the samples were annealed at a given temperature for 2 h before the measurements. The TTCA-I crystals at 160 ^{o}C and 245 ^{o}C revealed identical triclinic structures with $P\bar{1}$ space group having the unit cell parameters a = 5.587 Å, b = 7.047 Å, c = 8.799 Å, α = 102.99°, β = 92.87° and γ = 110.47°.

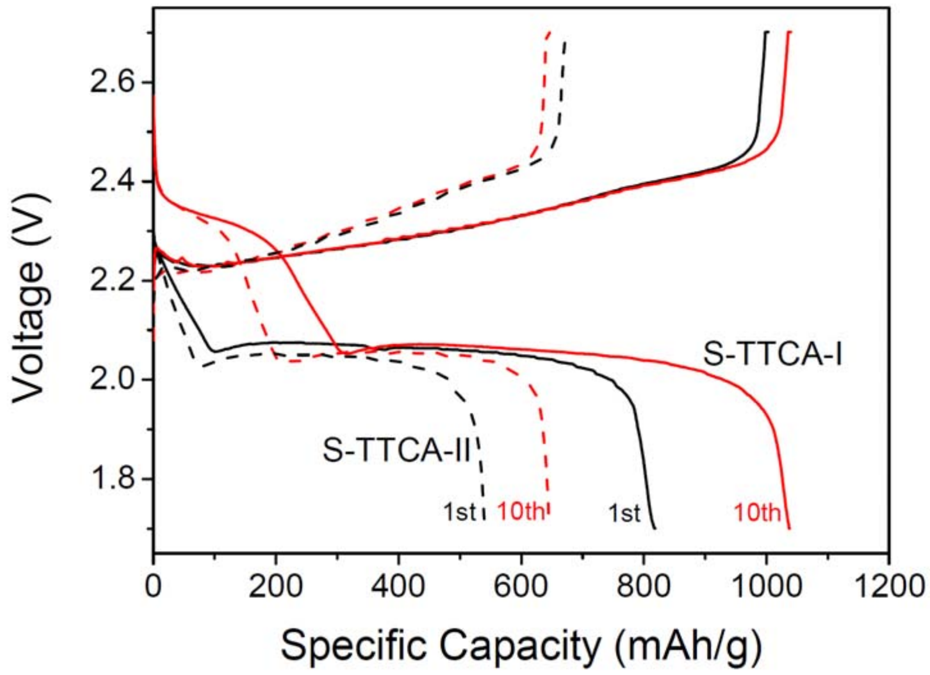

Supplementary Fig. 5. Battery performance of Li/S-TTCA-I and Li/S-TTCA-II cells. Representative galvanostatic discharge/charge voltage profiles of Li/S-TTCA-I (solid lines) and Li/S-TTCA-II (dashed lines) cells, cycled between 1.7–2.7V at 0.2C. Overall, the voltage profiles of the Li/S-TTCA-II cell bear resemblance to the Li/S-TTCA-I cell in their absence of the ring opening plateau at 2.33 V during the first discharge cycle. After the first discharge/charge cycle, two stable discharge plateaus appeared at 2.33 and 2.06 V for both Li/S-TTCA-I and Li/S-TTCA-II cells, ascribed to the appearance of S_8 after electrochemical scission and regeneration of disulfide bonds with cycling. The fact that Li/S-TTCA-II cell exhibited significantly lower specific capacities than Li/S-TTCA-I cell is noteworthy.

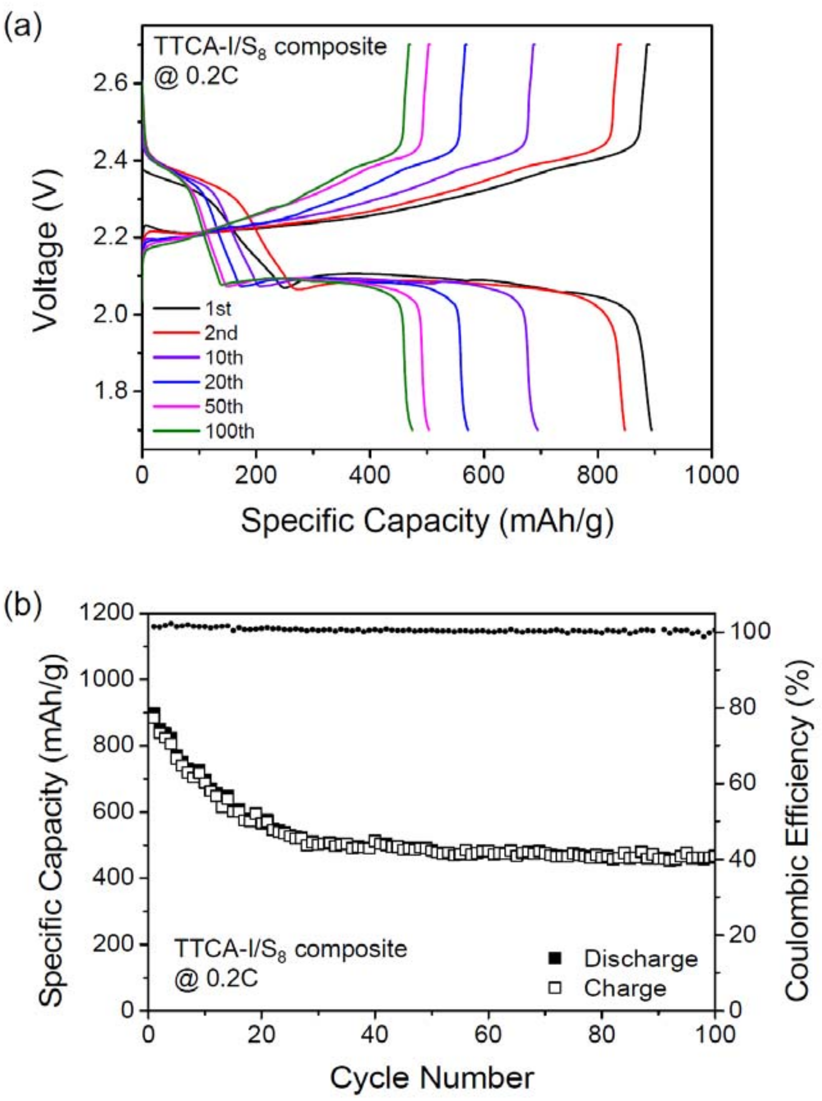

Supplementary Fig. 6. Battery performance of porous TTCA-I/S composite cathode. (a) Representative galvanostatic discharge/charge voltage profiles and (b) discharge/charge capacities and Coulombic efficiencies of the Li-S cells based on porous TTCA-I/sulfur composite cathodes for 100 cycles at 0.2 C. The composite cathodes were prepared by impregnating sulfur into porous TTCA frameworks without vulcanization reaction. Rapid capacity loss incurred during the initial 20 cycles for the porous TTCA-I/S composite cathode, which bears resemblance to S-C cathode rather than S-TTCA-I cathode. In addition, two plateaus appeared during the first discharge, in contrast to one plateau seen for the S-TTCA-I.

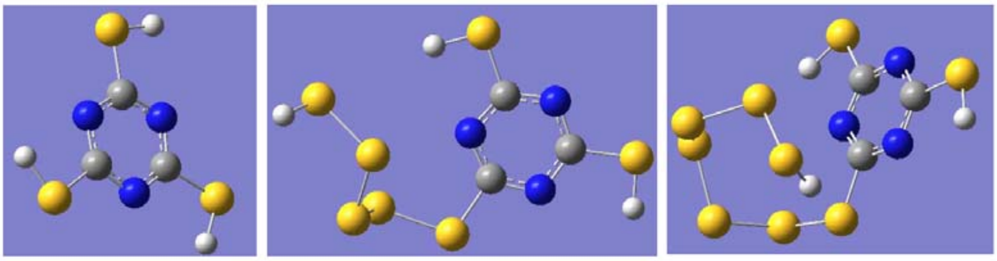

	TTCA	TTCA-S4	TTCA-S6
Band gap (eV)	5.76	4.84	4.59
LUMO (eV)	-1.59	-2.35	-2.53
HOMO (eV)	-7.35	-7.19	-7.12

Supplementary Fig. 7. HOMO-LUMO gaps of TTCA and S-TTCA. Quantitative investigation on the electronic structures of TTCA and S-TTCA by *ab initio* calculations at 0 K in a vacuum using a DFT exchange-correlation functional. The covalent attachment of sulfur to TTCA molecules resulted in a large decrease in band gap, attributed to a stabilized lowest occupied molecular orbital (LUMO) with decreased energy. For the neat TTCA, the band gap of 5.76 eV was determined at the B3PW91/6-31G** level, which decreased to 4.84 eV and 4.59 eV as a result of S_4 and S_6 attachment, respectively.

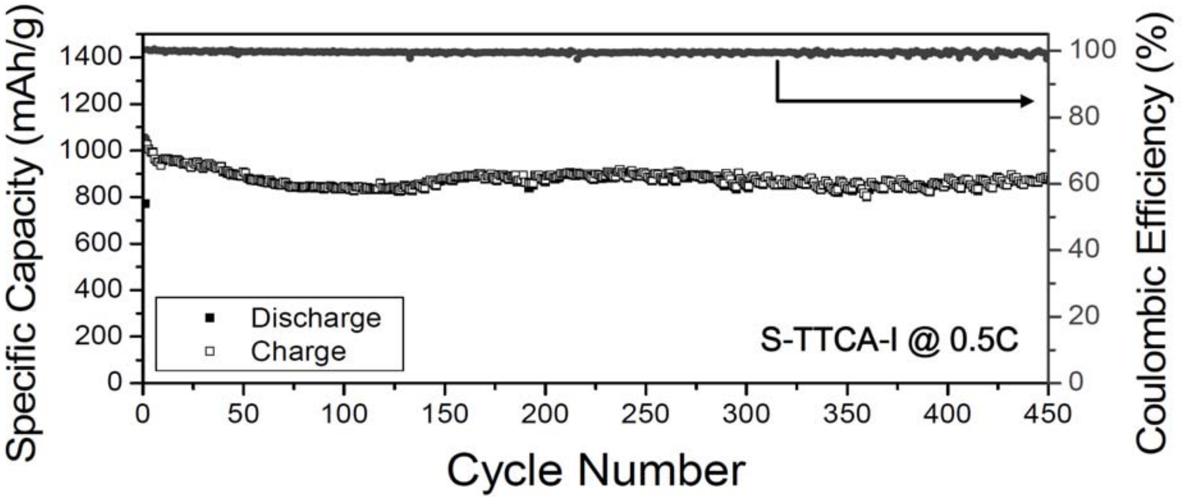

Supplementary Fig. 8. Long cycle life of Li/S-TTCA-I cells. Discharge/charge capacities and Coulombic Efficiencies of the Li/S-TTCA-I cells at 0.5 C with an extended life of 450 cycles. The cell was found to deliver a reversible capacity of 850 mAh/g after 450 cycles (corresponding to 83% capacity retention) with high Coulombic efficiency of 99%.
